# A multi-step screening algorithm for fecal microbiota transplantation donors: integrating metagenomic and metabolomic profiling for improved safety and donor quality assessment

**DOI:** 10.3389/fmicb.2026.1927788

**Published:** 2026-09-09

**Authors:** Alina V. Gospodarik, Nikolay I. Khromykh, Natalia D. Prokhorova, Yaroslav D. Shansky, Irina Balazs, Julia A. Bespyatykh

**Affiliations:** 1 Center of Molecular Medicine and Diagnostics, Lopukhin Federal Research and Clinical Center of Physical-Chemical Medicine of Federal Medical Biological Agency, Moscow, Russia; 2 Center for Bio- and Medical Technologies, Moscow, Russia; 3 Mendeleev University of Chemical Technology of Russia, Moscow, Russia

**Keywords:** fecal donor screening and selection, fecal microbiota transplantation (FMT), short-chain fatty acids (SCFAs), stool banking, stool microbiome

## Abstract

**Background:**

Fecal Microbiota Transplantation (FMT) has emerged as a highly effective treatment for recurrent *Clostridioides difficile* infection. It is also a promising therapeutic approach for other microbiome-related disorders. However, the safety and efficacy of FMT depend critically on rigorous donor screening and selection protocols. In this study, we developed and evaluated an optimized donor screening and selection workflow that integrates multistep pathogen detection, metagenomic and metabolomic profiling, and clinical compatibility assessment to enhance FMT safety and donor quality assessment.

**Methods:**

In this prospective cross-sectional study we evaluated 178 stool donor candidates using a novel screening algorithm, which combined extended medical history, blood and urine testing, stool metagenomics (16S rRNA gene sequencing) and metabolomics (short-chain fatty acids), and microbiological stool tests to exclude the stool samples with pathogens and ensure their microbial diversity.

**Results:**

The FMT donor screening and selection workflow was developed and included four main steps: 1. Potential donors (*n* = 178) had to meet all the study inclusion criteria (47.8% of donors passed this screening step); 2. Potential donors (*n* = 85) underwent microbiological and microscopic stool examination (23% of initially enrolled donors passed this screening step). Most of the healthy donors were excluded at this selection step due to the abnormal values of *Enterococcus spp*. (47.1% of healthy donors had abnormal values), total number of *Enterobacteriaceae* and *Bifidobacterium spp*. (for both parameters 41.2% of healthy donors had abnormal values) abundance; 3. Potential donors (*n* = 41) underwent blood (total blood cell count, biochemical blood analysis) and urine (urinalysis) tests. Only 13 donors (7.3% of the initial cohort) proceeded to the final metabolomic analysis, resulting in the selection of 3 FMT donors (1.7% eligibility rate).

**Conclusions:**

This study establishes a data-driven, high-stringency donor selection framework that provides a rational basis for improving FMT safety and donor quality. Our findings advocate for the standardized implementation of advanced screening technologies, particularly SCFA metabolomics, as a critical quality control step in stool banking. While clinical validation of the efficacy-enhancing potential of this protocol requires prospective studies, the strong association between butyrate-producing microbiota and favorable FMT outcomes, combined with our previous clinical observations, supports the utility of our approach.

## 1 Introduction

The human gastrointestinal tract (GIT) is colonized by numerous species of microorganisms that perform protective, metabolic, immunomodulatory, and nutritional functions, contributing to the maintenance of homeostasis. The gut microbiome composition is relatively stable and resilient but varies among individuals. The gut microbiome composition can be influenced by many factors including environmental factors, diet, viruses, medications, particularly antibiotics, etc. Many diseases are found to be associated with gut microbiome dysbiosis, including infections (e.g., infectious gastroenteritis, *Clostridioides difficile* infection), autoimmune diseases [e.g., allergic diseases, diabetes, inflammatory bowel disease (IBD)], and metabolic disorders (e.g., obesity, functional gastrointestinal disorders) (Shen et al., 2025). Therefore, it is of utmost importance to search for the ways to restore the healthy gut microbiome in patients suffering from gut dysbiosis associated conditions.

Several approaches to combat dysbiosis are currently suggested, including probiotics, prebiotics and fecal microbiota transplantation (FMT). Among those, FMT is shown to be most effective at restoring healthy microbiome composition being at the same time a relatively safe procedure with no significant long-term side effects (Goloshchapov et al., 2019). FMT has emerged as a highly effective treatment for recurrent *Clostridioides difficile* infection (rCDI) and is being investigated for other conditions linked to gut dysbiosis, such as inflammatory bowel disease, metabolic disorders, and neurological conditions.

FMT is most established for rCDI, with cure rates exceeding 90% in multiple randomized controlled trials (RCTs) (van Nood et al., 2013; Hvas et al., 2019). The FDA has approved FMT for rCDI under enforcement discretion. FMT is proven to be an effective treatment for recurrent *Clostridioides difficile* infection and is officially approved by the U.S. Food and Drug Administration (FDA) for the treatment and prevention of recurrent gastrointestinal infection caused by vancomycin-resistant strains of *C. difficile* (Rebyota, Vowst) (Akinshina et al., 2019; Vasilyev et al., 2015; Gulati et al., 2023).

Apart from rCDI FMT is proposed as a treatment strategy for the Inflammatory Bowel Disease (IBD), although the studies show mixed results. For example, in ulcerative colitis FMT is shown to be effective (Feng et al., 2023) with some RCTs reporting remission in −25–30% of patients (Paramsothy et al., 2017). However, FMT application in Crohn's Disease has limited evidence with variable responses reported (Sokol et al., 2020). In Irritable Bowel Syndrome (IBS) preliminary studies suggest symptom improvement upon FMT, particularly in diarrhea-predominant IBS (IBS-D) (Johnsen et al., 2018).

The success of FMT largely depends on the quality and safety of the donor derived microbiota, making rigorous donor screening and selection a critical component of the process.

FMT donor screening aims to minimize the risk of transmitting infectious diseases, antibiotic-resistant microorganisms, and other potential health risks while ensuring the donor's microbiome is diverse and beneficial. Current protocols include comprehensive medical history assessments, laboratory testing for pathogens, and evaluation of stool microbiome composition. However, there is no standardization across institutions regarding the FMT donor selection algorithm (Liu et al., 2025). Therefore, the development of the standardized approach for FMT donor selection process is of utmost importance to enhance FMT efficacy and safety.

This study explores strategies to optimize the donor screening and selection workflow for FMT, incorporating advances in microbial profiling, biomarker identification, and standardized exclusion criteria. We evaluate the impact of multi-modal screening approaches including comprehensive medical history assessments, advanced stool testing, and metagenomic sequencing on FMT donor eligibility. Furthermore, we propose a streamlined, evidence-based framework to enhance donor selection efficiency while maintaining stringent safety standards. By refining these protocols, we aim to improve the reliability, scalability, and therapeutic success of FMT in clinical practice. While the clinical efficacy of FMT for recurrent CDI and other conditions has been established in our previous studies (Seregin et al., 2024; Bespyatykh et al., 2023) and by others (Hvas et al., 2019), the critical determinants of donor quality that predict successful engraftment and therapeutic response remain poorly characterized. This study does not aim to reassess clinical efficacy, but rather to establish a rigorous, data-driven, and reproducible framework for donor selection. We hypothesize that integrating high-resolution microbial profiling with functional metabolomics (SCFAs) into the screening workflow will enhance the identification of ‘optimal' donors, thereby potentially maximizing the clinical benefits observed in our prior trials.

## 2 Materials and methods

### 2.1 Human subjects

This prospective cross-sectional study was designed to develop and validate an optimized donor screening and selection workflow, rather than to evaluate the therapeutic efficacy of FMT itself. The clinical outcomes of FMT using material from donors selected through a similar (but previous version of) protocol have been reported elsewhere (Seregin et al., 2024; Bespyatykh et al., 2023).

Healthy volunteers (*n* = 178) were recruited at the Department of Molecular Medicine of Lopukhin Federal Research and Clinical Center of Physical-Chemical Medicine of Federal Medical Biological Agency between 2022 and 2024 within clinical study (registration number: NCT04011943). All healthy volunteers provided written informed consent, and the study was approved by the Lopukhin Federal Research and Clinical Center of Physical-Chemical Medicine of Federal Medical Biological Agency Institutional Review Board (ethic vote number: 2022/05/31) and carried out according to the Declaration of Helsinki and Good Clinical Practice guidelines. Presence of active or chronic infection, inflammatory bowel disease (IBD), constipation or diarrhea within 3 weeks prior, tattooing or piercing within 6 months prior, autoimmune disease, atopic disease or allergic reaction, metabolic syndrome, malignancy, alcohol intake or smoking within 3 weeks prior, treatment with narcotic or psychotropic drugs or other toxic substances, hormones or antibiotics within 6 months prior, absence of signed informed consent were exclusion criteria. Healthy volunteers were included into the study if they were between 18 and 50 years old with body mass index (BMI)-−18, 5–24,99 kg/m^2^. Stool, blood and urine samples were collected and detailed medical history was obtained. Stool and urine samples were collected into sterile collection tubes either on the same day or in the evening before the study visit. Blood samples were collected after overnight fasting. Full blood count, total bilirubin, direct bilirubin, glucose, creatinine, urea, aspartate aminotransferase (AST), alanine aminotransferase (ALT), alkaline phosphatase (AP), alpha-amylase were measured in the blood, as well as the tests for the hepatitis B, hepatitis C, HIV, syphilis infections were performed. Urine was collected for urinalysis. Stool samples were collected for analysis of gut microbiome composition, coprogram, occult blood test, protozoa and helminths, pathogenic intestinal flora (*Salmonella, Shigella, Campylobacter, enteroinvasive Escherichia coli, Adenovirus F, Rotavirus A, Norovirus (type 2), Astrovirus)*, presence of SARS-CoV-2 virus and drug resistance genetic markers (VIM, NDM, OXA-48, KPC, MecA/ErmB, Mef / ErmB, VanA / VanB, TEM, CTX-M).

### 2.2 Stool culture test

Stool culture test was carried out in accordance with the guidelines “Bacteriological diagnostics of dysbacteriosis” (Epstein-Litvak R. V., Vilshanskaya F. L. Bacteriological Diagnosis of Intestinal Dysbacteriosis. Methodological Recommendations. Moscow, 1977; 20 p.). In addition, an extended analysis was carried out for lactobacilli using solid differential nutrient media and biochemical identification cards of the VITEK^®^ 2 Compact microbiological analyzer (bioMérieux, France).

In brief, one gram of stool sample was homogenized in 9 ml of sterile saline and left at room temperature for 15 min. The resulting suspension and its serial dilutions were plated on differential nutrient media to detect different clinically relevant species of microorganisms (Table 1).

**Table 1:** Clinically relevant species of microorganisms detected with the stool culture test.

Dilution	Microorganism	Medium	Test results evaluation
10^−8^, 10^−7^	Bifidobacteria	Semi-liquid nutrient medium Blaurock (FBUN GNC PMB Obolensk, Russia)	After 72 h
10^−7^	Lactobacilli	Semi-liquid nutrient medium MRS-2 (FBUN GNC PMB Obolensk, Russia)	After 72 h
10^−5^	Clostridia	1 ml deep plated on iron sulfite agar (Biokompas-S; Russia)	After 72 h
10^−5^	Hemolytic bacterial species	Blood agar (HiMedia Laboratories; India)	After 20–22
10^−5^	Gram-negative Enterobacteria	Endo medium (HiMedia Laboratories; India)	After 20–22 h
10^−3^	Non-fermenting gram-negative bacteria	Endo medium (Biokompas-S; Russia)	After 20–22 h
10^−3^	Pathogenic fungi	Sabouraud medium (Biotechnovatsiya; Russia)	After 48 h
10^−3^	Staphylococci	Yolk-salt agar (HiMedia Laboratories; India)	After 48 h
10^−3^	Enterococci	Milk-inhibitory medium (“Biokompas-S”; Russia)	After 48 h
10^−1^	Pathogenic Enterobacteria	SS- and XLD-agar (HiMedia Laboratories, India)	After 20–22 h
10^−1^	Pathogenic *E.coli*	selenite broth (Biocompas-S, Russia)	After 24 h

### 2.3 PCR analyses

Total stool DNA was extracted with DNA-SORBENT kit (Litech, Russia) or Ribo-Sorb kit (AmpliSense, Russia) according to the manufacturer's instructions. Analysis of genetic markers of drug resistance were performed with the following kits: Resistome.CTX-M, Resistome.MecA, Resistome.OXA10, Resistome.DHA, Resistome.VIM, Resistome.NDM, Resistome.OXA48, Resistome.KPC, Resistome.blaGES, Resistome.OXA40-like, Resistome.Van, Resistome.ErmB, Resistome.Mef (all kits were from VITA-R.O.S., Russia).

Analysis of *Clostridioides difficile* and toxins A and B gene abundance was performed with the Triplex CDT kit (Litech, Russia). The presence of SARS-CoV-2 infection was detected with the Polyvir SARS-CoV-2 kit (Litech, Russia). *Salmonella, Shigella, Campylobacter, Enteroinvasive Escherichia coli, Adenovirus F, Rotavirus A, Norovirus (type 2), Astrovirus* presence were detected with the AmpliSense ^®^ AII screen-FL kit (AmpliSense, Russia).

### 2.4 Stool SCFAs

Stool samples were weighed and −0.18 g was placed in an Eppendorf tube. 0.5 M hydrochloric acid solution was added up to 1.8 g and mixed on a Vortex stirrer (ELMI, Latvia) for 4 h. Then the tube with the sample was centrifuged at 3,000 rpm for 5 min (Eppendorf 5480R, Germany), and 0.5 ml of supernatant was transferred to another tube. 0.45 ml of HPLC-grade acetonitrile (VWR, the Netherlands) and 0.05 ml of dioxane (internal standard; Khimmed, Russia) were added. The tube was vortexed and centrifuged at 13,200 rpm for 15 min. The resulting supernatant was further analyzed.

Qualitative and quantitative analysis of SCFAs due to their volatility was performed by gas-liquid chromatography with flame ionization detection. SCFAs were separated using a gas chromatograph “Crystal 2000M” (Chromatek, Russia). 1 μl of the supernatant was introduced with a microsyringe (Hamilton, Switzerland) into the evaporator of the chromatograph equipped with a Stabilwax-DA column (Restek, USA) l.30 m, i.d. 0.33 mm and the flame ionization detector. The stationary phase was polar (copolymer of Carbowax polyethylene glycol with terephthalic acid film, 0.34 micr.). The mobile phase was nitrogen with an inlet pressure of 1.8 atm at the column inlet. Carrier gas consumption was 2.0 ml/min, air was 300 ml/min. The mode was split, 50:1. Analysis time was 20 min. Operating mode was a gradient with thermostat temperature 80°C (2 min) → 220°C at heat rate 10°C/min. Inject and detector temperatures were 220°C.

The following indicators were determined using the internal standard method: the content (in μg/g) of acetic acid (C2), propionic acid (C3), butyric acid (C4), isobutyric acid (iC4), valeric acid (C5), isovaleric acid (iC5), caproic acid (C6) acids, their total absolute abundance, anaerobic index (AI) and the ratio of the total abundance of branched-chain acids (isomers) to the total abundance of straight-chain acids (isoCn/Cn).

### 2.5 16S rRNA sequencing

Stool DNA isolation. In brief, 400 μL of PBS were added to the 50 mg of stool sample, which were then homogenized with glass beads using a MagNA Lyzer device (Roche, USA) for 30 s. For further DNA extraction a King Fisher Flex automatic station (Thermo Fisher Scientific, USA) using a MagMAX™ Microbiome Ultra Nucleic Acid Isolation Kit (Thermo Fisher Scientific, USA) were used in accordance with the manufacturer's protocol. The extracted DNA concentration was measured with Qubit fluorometer with the Quant-iT dsDNA Broad-Range Assay Kit (Thermo Fisher Scientific, USA). 16S rRNA gene was amplified with Tersus Plus PCR kit (Evrogen, Russia) using standard primers 27F (AGAGTTTGATYMTGGCTCAG) and 1492R (GGTTACCTTGTTAYGACTT) (Evrogen, Russia) and 5ng of DNA template. The following amplification program was used: 95°C-−2 min; (95°C-−1 min; 60°C-−1 min; 72°C-−3 min;) 30 cycles; 72°C-−2 min. PCR products were checked with the gel electrophoresis (1.5% agarose gel) and were purified with KAPA Pure Beads magnetic beads (Roche, USA) in accordance with the manufacturer's protocol.

DNA libraries were prepared with NEBNext^®^ Ultra™ II End Repair/dA-Tailing Module kit (NEB) and ligated with Native Barcoding Kit 96 (SQK-NBD109.96) using Blunt/TA Ligase Master Mix (NEB) according to the manufacturer's instructions. Barcoded libraries were purified using KAPA Pure Beads (Roche, USA). Library concentrations were measured with the Quant-iT dsDNA Assay Kit, High Sensitivity (Thermo Fisher Scientific, USA), and the samples were mixed in equal molar concentrations. The final adapter (Adapter Mix II Expansion (Oxford Nanopore Technologies, UK)) was ligated to the pooled library using NEBNext Quick Ligation Module (NEB). Sequencing was performed with the MinION Mk1B device (Oxford Nanopore Technologies, UK). The resulting sequencing data were analyzed with the bioinformatics pipeline which was implemented using Snakemake (v7+). Adapter sequences were identified and removed using Porechop (v0.2.4). To retain only high-quality 16S rRNA gene amplicons, the trimmed reads were processed using Chopper (v0.6.0). Reads were filtered based on a minimum Phred quality score of 10 and a length threshold. Specifically, reads shorter than 1,300 bp and longer than 1,600 bp were discarded, selecting for full-length or near-full-length 16S rRNA gene sequences. To assess the yield and quality of the sequencing data before and after pre-processing, NanoStat (v1.6.0) was executed on the raw and filtered FASTQ files. Taxonomic assignment and relative abundance quantification were performed using EMU (v3.4.5), which aligns reads against a reference database using minimap2 (v2.26) in map-ont mode, specifically tailored for long, noisy nanopore reads. EMU uses a maximum likelihood approach to map reads to a 16S rRNA reference database (Emu prebuilt reference database with Silva 138.2). The resulting mean sequencing depth was 5,692 reads per sample. For alpha diversity assessment, data were rarefied and Shannon and Simpson indices were calculated to quantify microbial diversity using vegan package. Beta diversity was done on an unrarefied feature table after total sum scaling. Beta diversity was calculated using non-metric multidimentional scaling based on Bray–Curtis distance.

### 2.6 Statistical analysis

Statistical analysis and data visualization were performed with R (2023.06.0 + 421, Posit Software, USA) and IBM SPSS Statistics 27 (developed by IBM, USA). For SCFA data, the Mann-Whitney U-tests was used to compare the groups. For descriptive statistics of SCFA data, median and interquartile range (IQR) are reported. Categorical variables in Tables 2, 3 are presented as frequencies and proportions without inferential testing. *P* < 0.05 was considered statistically significant.

**Table 2:** The results of the microbiological stool test.

Microflora	Normal values, CFU/g	Number of healthy donors, n	
		Normal values	Deviation from the normal values
*Bifidobacterium spp*.	10^8andhigher^	50	35
*Lactobacillus spp*.	10^6^−10^7^	70	15
Total number of *Enterobacteriaceae*	10^7^−10^8^	50	35
*Escherichia spp*.	10^7^−10^8^	56	29
*Enterococcus spp*.	10^6^−10^7^	45	40
Opportunistic *Enterobacteriaceae* bacteria: *Enterobacter cloacae, Enterobacter gergoviae, Citrobacter freundii, Citrobacter amalonaticus*.	< 10^4^	65	20
Pathogenic microorganisms of *Enterobacteriaceae* family	shouldn't be	85	0
*Staphylococcus spp*.	< = 10^4^/shouldn't be present	78	7
*Staphylococcus aureus*	< = 10^4^/shouldn't be present	78	7
*Candida* spp.	< = 10^4^	83	2
Non-fermenting gram-negative bacilli including *Pseudomonas aeruginosa, Pseudomonas putida*	< = 10^4^	82	3
Anaerobic sulfite-reducing bacteria genus *Clostridioides*	< = m10^6^	76	9

**Table 3:** The results of coprogram and occult blood test.

Parameter	**Number of healthy donors**, ***n***	
	Normal values	Deviation from the normal values
Fatty acids	60	25
Iodophilic flora	75	10
Hidden blood	83	2
Mucus	82	3
Crystals	83	2
Indigestible plant fiber	80	5

## 3 Results

### 3.1 Patients' recruitment, selection and characteristics

**Figure 1:**
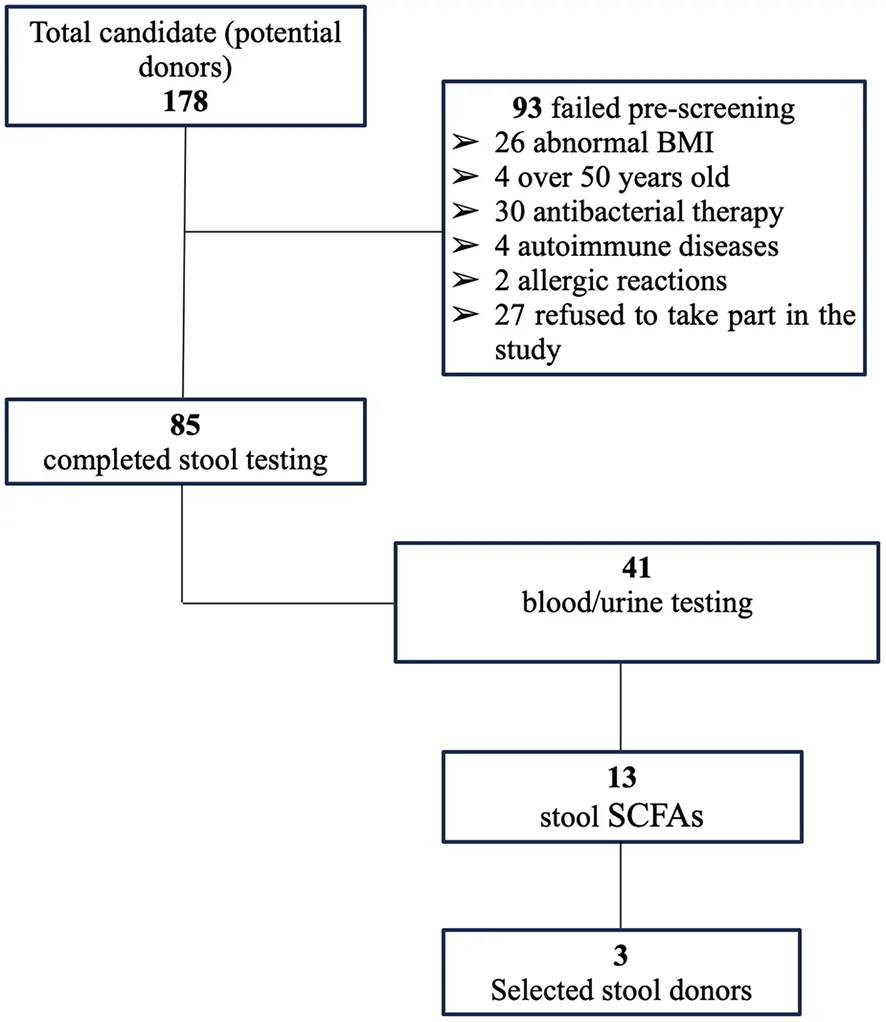
Flow diagram of donor screening and selection process. The diagram shows the number of candidates at each screening step and the reasons for exclusion.

178 potential stool donors were recruited via posters, flyers and social networks (year 2022 to 2024) (male *n* = 59, female *n* = 119). 93 potential donors (44.9 %) did not meet the inclusion criteria of the study. 26 potential donors (14.6%) had abnormal body mass index (BMI), 4 potential donors (2.2%) were over 50 years old, 30 potential donors (16.7%) received antibacterial therapy within last 6 months prior to the study, 4 potential donors (2.2%) had confirmed autoimmune disease diagnosis, 2 potential donors (1.1%) had a medical history of allergic reactions and 27 potential donors (15.1 %) did not sign the informed consent (Figure 1). 85 potential healthy donors (male *n* = 32, female *n* = 53) met all the inclusion criteria and were enrolled into the study.

Laboratory examination (stool, blood and urine tests) is an integral part of the entire donor selection process to exclude risks for the potential transmission of infectious pathogens from donor to recipient. Laboratory tests used in the second round of healthy donor screening included microbiological stool analysis (*n* = 85) and coprogram analysis (*n* = 85). 44 potential stool donors did not meet the inclusion criteria at this donor selection step. 41 potential stool donors entered the third round of the selection process, which included complete blood count, biochemical analysis and urinalysis. 34 potential stool donors did not meet the inclusion criteria of the third round of the donor selection process. 13 donors (7.3% of initially screened potential stool donors) were included into the fourth step of the FMT donor selection workflow. After stool SCFAs levels analysis only 5 healthy donors (donor eligibility rate 1.7%) were selected as FMT donors (Figure 1). However, 2 of these donors refused to futher participate in the study and therefore the stool microbiota analysis with 16S rRNA sequencing was performed for only 3 remaining selected FMT donors (donor eligibility rate 1.7%) to obtain detailed data about their microbiota structure and function. So, final selected donors (*n* = 3) — donors who passed all screening steps and were chosen based on SCFA profiling and rejected donors (*n* = 10) — donors who passed the third step (blood/urine) but were not selected for the final stage due to lower SCFA levels or because they refused to further participate in the study.

### 3.2 Microbiological analysis of stool samples

The qualitative and quantitative evaluation of the microbiological composition of fecal samples is one of the key criteria for FMT donor selection. In the study, a microbiological assessment of stool samples from potential healthy donors (*n* = 85) was conducted at the second step of the donor selection workflow (Table 2).

The highest deviation from the reference levels was detected for *Enterococcus spp*. (47.1% of healthy donors had abnormal values), total number of *Enterobacteriaceae* (41.2% of healthy donors had abnormal values), and *Bifidobacterium spp*. (41.2% of healthy donors had abnormal values).

The abundance of obligate microbiome species, such as: *Bifidobacterium, Lactobacillus*, and *Escherichia* were within the normal range for 58.8%, 82.4%, and 65.9% potential FMT donors respectively, while *Enterococcus* was within the normal range for 52.9% potential donors.

Opportunistic pathogenic bacteria were detected in the stool samples of some of the healthy donors and were represented by species like *Enterobacter cloacae, Staphylococcus aureus, Citrobacter freundii, Citrobacter amalonaticus, Pseudomonas aeruginosa, Pseudomonas putida*, and *Enterobacter gergoviae* (Table 2).

Pathogenic microorganisms of *Enterobacteriaceae* family, *Candida* spp, nonfermenting gram-negative bacilli including *Pseudomonas aeruginosa, Pseudomonas putida* were within the normal values in the majority of screened for eligibility individuals.

Thus, the stool culture analysis revealed that only 23 % (*n* = 41) of the initially recruited potential healthy donors met the inclusion criteria based on the microbiological stool test results.

### 3.3 Coprogram

As a next step coprogram as well as occult blood test were performed to exclude healthy donors with the digestive tract dysfunction (*n* = 85) (Table 3).

43 healthy donors had coprogram parameters within the reference values, which accounted for 24.2% of initially enrolled healthy donors. Out of these 43 healthy donors 2 donors had a positive result in the occult blood test and therefore were excluded from the next steps of the study. It is worth mentioning that almost 30% of healthy donors had an elevated level of stool fatty acids, but almost all the healthy donors had such parameters as mucus, crystals, hidden blood within the normal values (Table 3).

### 3.4 Blood tests

Total blood count and biochemical blood analyses were performed for 41 potential FMT donors. Deviations from the normal blood parameters were found in 34 (19.1 % of initially recruited healthy donors) study participants. Specifically, deviations in the leukocyte formula were detected in 9 (5.1% of initially recruited healthy donors) participants. This might indicate the potential presence of systemic diseases, allergies, endocrine dysfunction, inflammatory processes in these healthy donors. According to the results of the biochemical blood analysis, deviations from the reference values were observed in only 5 (2.8% of initially recruited healthy donors) study participants, mainly an elevation in the levels of total bilirubin and alanine aminotransferase (ALT). This might indicate the presence of liver or cardio-vascular system disorders in these study participants.

### 3.5 Stool SCFAs

**Figure 2:**
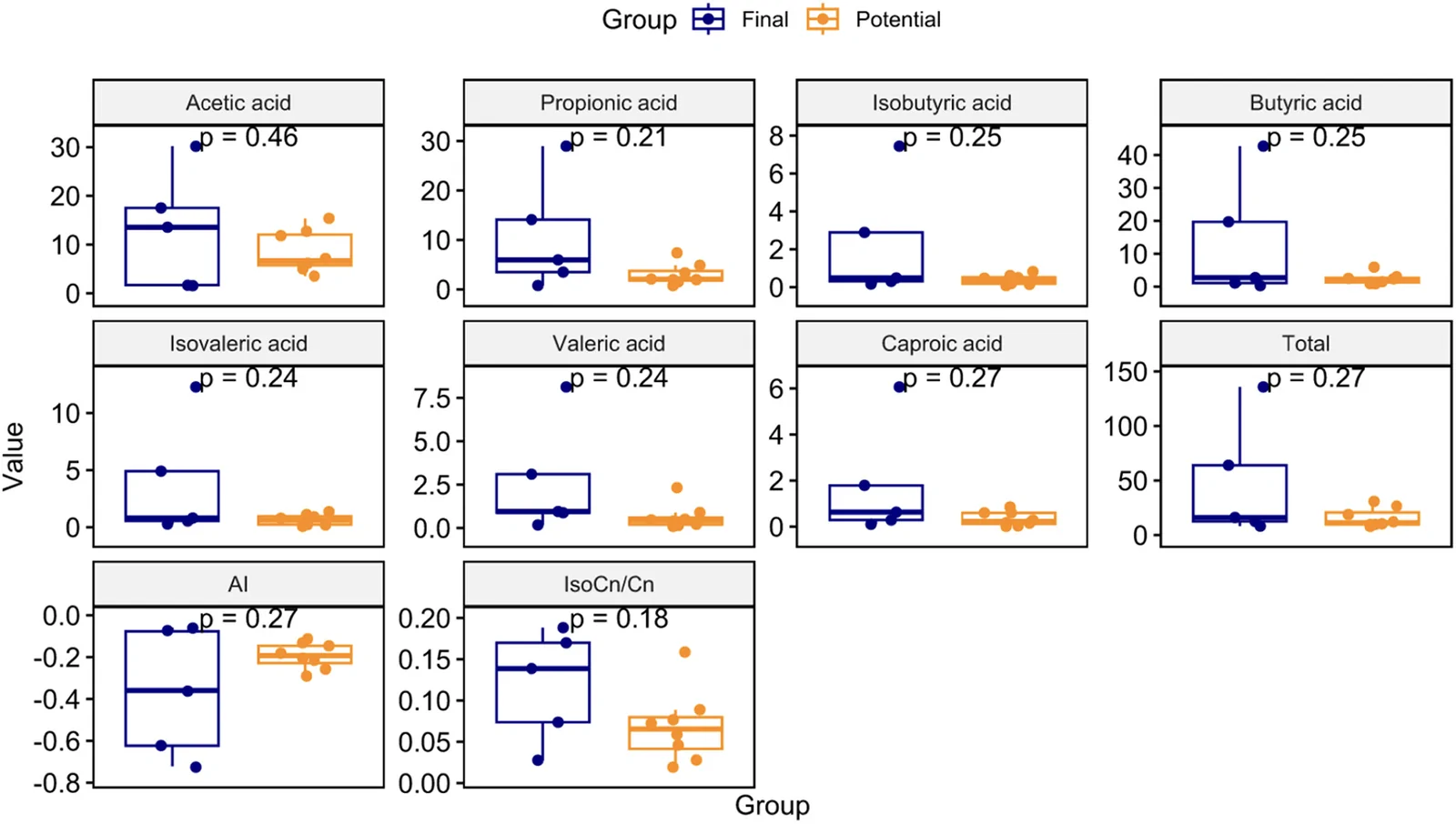
Comparison of short-chain fatty acid (SCFA) concentrations (μg/g) between selected (*n* = 5) and rejected (*n* = 8) fecal microbiota donors. The raw data (circles), median (horizontal line), interval Q1–Q3 (box), and interval without outliers (whiskers) are presented. C2, acetic acid; C3, propionic acid; iC4, isobutyric acid; C4, butyric acid; iC5, isovaleric acid; C5, valeric acid; C6, caproic acid; Total, total SCFAs. No statistically significant differences were observed between groups (Mann-Whitney *U*-tests, *p*> 0.05 for all comparisons).

SCFAs were measured in the stool samples from healthy donors who passed the third screening step (blood and urine tests) (*n* = 13) (Figure 2). Among these, 5 donors were selected as optimal FMT donors based on the highest total SCFAs and butyric acid levels, while 8 donors were classified as rejected (i.e., they passed the third step but were not selected for the final stage due to lower SCFA levels). The comparison of stool SCFA levels between the selected (*n* = 5) and rejected (*n* = 8) groups is presented in Figure 2 and summarized below.

Total SCFAs mean level was 15.77 (median 11.22, Q1—Q3 9.75–20.35) in potential donors and 47.34 (median 16.11, Q1—Q3 12.51–64.03) in final donors (p 0.213). Therefore, the group of selected donors had almost 3 times higher total stool SCFAs levels, although it was not significant. The most abundant SCFA in the group of potential donors was acetic acid, with mean 8.47 (median 13.53, Q1–Q3 1.68-−17.52) μg/g, whereas in the group of selected donors it was butyric acid, with mean 13.31 (median 2.75, Q1–Q3 1.095-−19.710) μg/g. In both groups, the lowest level was observed for caproic acid. Its mean values were 0.34 (median 0.225, Q1–Q3 0.12-−0,60) μg/g in the group of potential donors, and 1.78 (median 0.63, Q1—Q3 0.29-−1,79) μg/g in the group of selected donors). There was no statistically significant difference between two groups for all studied SCFAs. This can be explained by the small sample size for this comparison. The difference between the groups in the parameters measured was observed for the anaerobic index, which means were −0.194 (median −0.193, Q1—Q3 −0.228– −0.146) in potential donors and −0.368 (−0.359, 0–0.623– −0.076) in final donors (p 0.270).

### 3.6 Stool microbiota analysis

**Figure 3:**
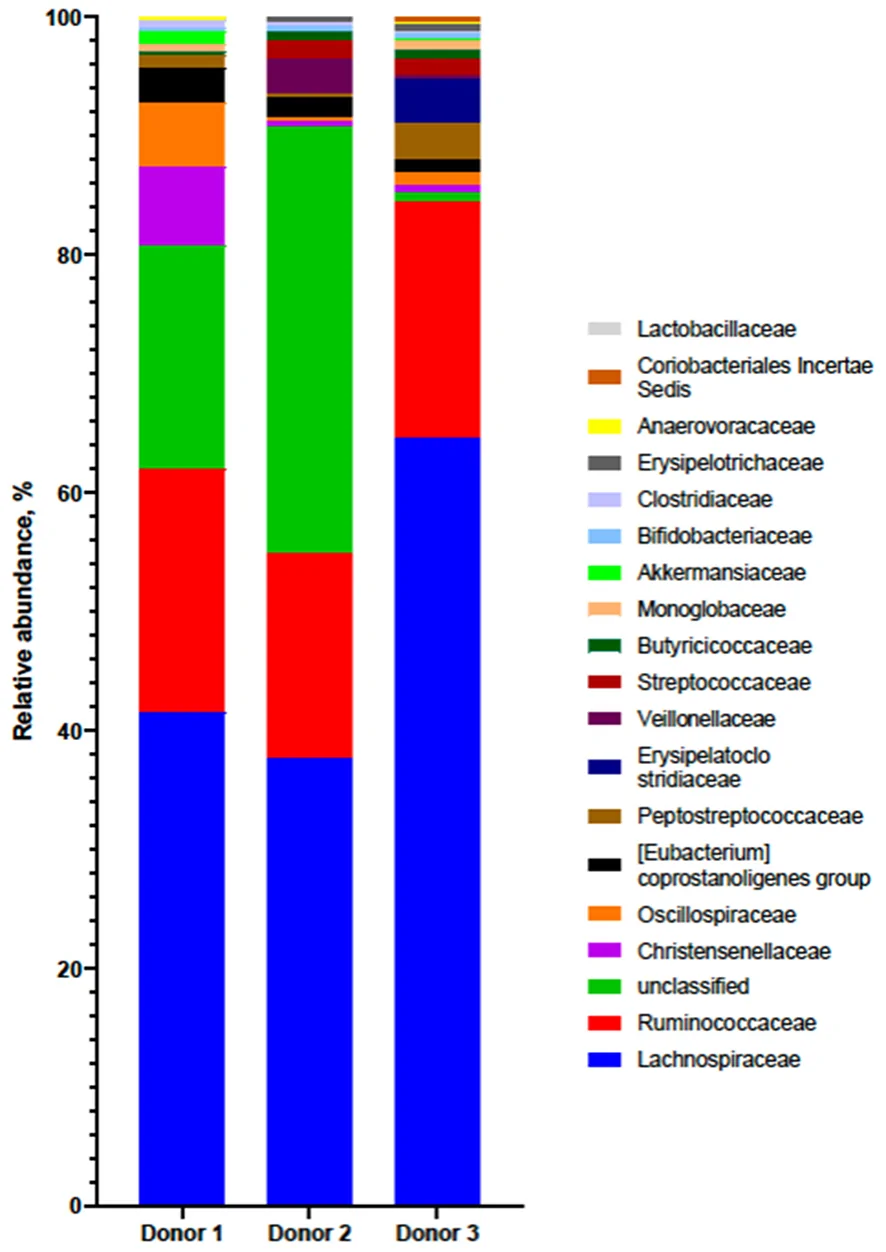
Results of 16S rRNA sequencing analysis of stool microbiota in three final FMT donors. Stool microbiota composition is visualized as relative abundances (%) of different bacterial groups at the family level, represented in a stacked bar chart.

16S rRNA gene sequencing was performed with the stool samples from 3 selected for FMT donors to examine their microbiota composition in detail (Figure 3).

The microbiota composition of all 3 donors was mainly represented with *Lachnospiraceae* (mean ± SD = 53.8 ± 11.1%) and *Ruminococcaceae* (mean ± SD = 19.1 ± 1.7%) bacterial families. Among the bacteria with the highest prevalence on the species level were *Blautia* sp. SC05B48*, Gemmiger formicilis, Anaerostipes hadrus* and *Faecalibacterium_prausnitzii*.

**Figure 4:**
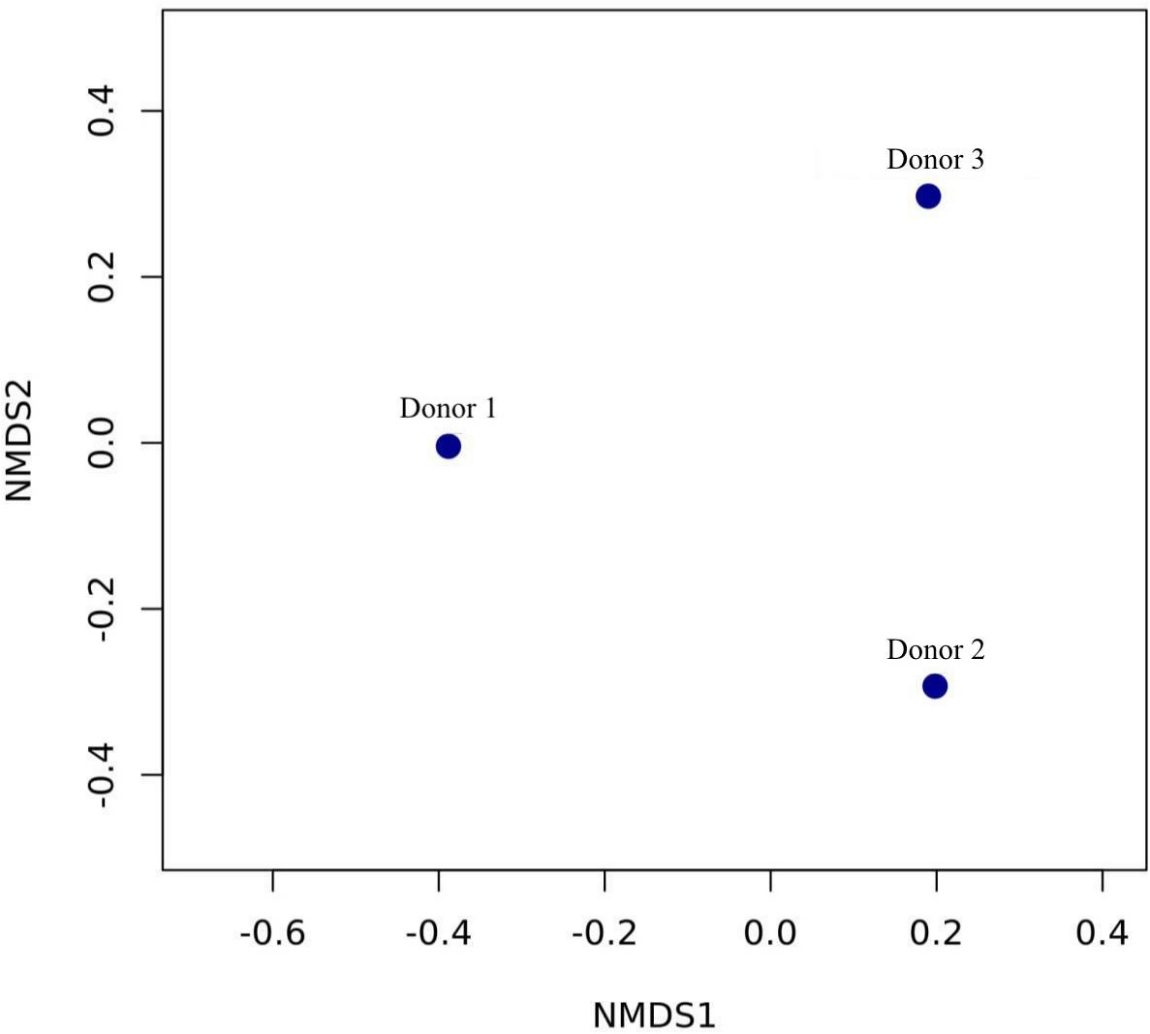
Non-metric multidimentional scaling (NMDS) plot based on Bray-Curtis dissimilarity showing beta diversity of the three final FMT donors.

Alpha diversity analysis of the three selected donors revealed Shannon indices ranging from 2.97 to 3.27 and Simpson indices from 0.913 to 0.938, indicating high species richness and evenness. Non-metric multidimentional scaling (NMDS) based on Bray-Curtis dissimilarity illustrates the microbial community structure of the 3 selected donors, showing their relative proximity in the sample space (Figure 4).

## 4 Discussion

FMT is recommended for the treatment of recurrent Clostridial infections, and in recent years, it has also been used to treat other diseases, not necessarily related to the gastrointestinal system. For instance, FMT has already been applied in patients with metabolic disorders. Animal and human studies indicated that FMT may improve insulin sensitivity and weight regulation (Kootte et al., 2017), but long-term efficacy remains uncertain. Emerging research explores the gut-brain axis, with small studies reporting behavioral improvements in autism spectrum disorder (Kang et al., 2017). The therapeutic effects of FMT are attributed to microbial restoration (replenishing beneficial bacteria that outcompete pathogens), immune modulation (influencing host immunity through microbial metabolites [e.g., short-chain fatty acids]), bile acid metabolism (altering bile acid profiles, which helps e.g. inhibiting *C. difficile* growth), colonization resistance (restoring gut barrier function and preventing pathogen overgrowth).

The therapeutic success of FMT is a multifactorial outcome, influenced not only by donor quality but also by recipient factors (e.g., disease severity, immune status, baseline microbiome), preparation methods, and route of administration. A direct causal link between our specific multi-parametric screening algorithm and enhanced clinical remission rates would require a large-scale, randomized controlled trial comparing our protocol against standard screening, which was beyond the scope of this investigation.

However, the strength of our study lies in its systematic, stepwise approach to eliminating suboptimal donors. We demonstrated that conventional screening (medical history, basic stool culture) alone results in a high false-positive rate (23% eligibility), necessitating deeper analyses. By adding SCFA profiling, we were able to select donors with a significantly enriched butyrogenic profile, which numerous studies have associated with improved colonization resistance and immune modulation (Bénard et al., 2024; Barnes et al., 2018; Wilson et al., 2019). Given that the donors selected through this enhanced workflow provided the material for our previous successful clinical case series (Seregin et al., 2024; Bespyatykh et al., 2023), we are confident that our algorithm represents a robust, evidence-based strategy for standardizing donor selection. Future prospective studies are warranted to quantitatively validate the superiority of this metabolomics-enhanced approach over conventional screening in a head-to-head clinical trial.

One of the biggest challenges currently is the selection of suitable stool donors for FMT. It however gets easier upon the establishment in many countries of the stool biobanks.

The success of FMT therapy is dependent on the comprehensive and rigorous screening of potential stool donors (Melikhov and Tsepova, 2017). The FMT starts with donor search and evaluation, following the preparation of fecal material, and its administration to the patient. Donor selection and screening are among the most critical aspects of FMT, as they must ensure the safety for the recipient. Donor screening typically includes questionnaires, physical examinations, and laboratory tests (Bibbò et al., 2020; Woodworth et al., 2017). The selection process of healthy volunteers who can become fecal microbiota donors involves several stages (Pavletic, 2020). First, volunteers who have no clinical signs of acute or chronic illness or risk factors and have results of lab and instrumental examinations within reference values are enrolled (Deiteren et al., 2021). These potential donors are allowed to pass the next donor screening stage if they fulfill the exclusion criteria, which typically include certain infectious diseases (HIV, viral hepatitis, syphilis, malaria, tuberculosis, zoonotic infections) or their risk factors (intravenous drug use, recent blood transfusions, tattoos, piercings, acupuncture, vaccination with live vaccines, etc.), non-infectious diseases (chronic kidney disease, cardiovascular diseases, obesity, mental and neurological disorders, autoimmune intestinal diseases, adherence to strict restrictive diets), and intake of certain medications (antibiotics, cytostatics, immunosuppressants, proton pump inhibitors) which can alter the composition of the gut microbiome (Cammarota et al., 2017). Potential donors must do standard laboratory tests including complete blood count, blood tests for cytomegalovirus, Epstein-Barr virus, hepatitis viruses (A, B, C, D, E), syphilis, HIV-1 and HIV-2, Entamoeba histolytica, C-reactive protein and erythrocyte sedimentation rate, albumin, creatinine and electrolytes, aminotransferases, bilirubin, gamma-glutamyl transferase, alkaline phosphatase; general fecal examination, including detection of *Clostridioides difficile*, intestinal pathogens including *Salmonella, Shigella, Campylobacter, Escherichia coli O157:H7, Yersinia spp*., vancomycin-resistant enterococci, methicillin-resistant *Staphylococcus aureus*, multidrug-resistant gram-negative bacteria, norovirus, antigens and/or acid-fast staining for *Giardia lamblia* and *Cryptosporidium parvum*, protozoa (including Blastocystis hominis), and helminths, fecal occult blood analysis, antigens and/or acid-fast staining for isopores and microsporidia, calprotectin, *Helicobacter pylori* fecal antigen, rotaviruses (Bénard et al., 2022). Furthermore, when selecting a donor for FMT, data from the donor and recipient's medical-genetic examination, as well as patient's immune status, may be taken into account (Chen et al., 2021).

A promising approach is to study and compare microbiome and metabolome composition of the donor and recipient's fecal microbiota (Porcari et al., 2023). For example, short-chain fatty acids (SCFAs) which are produced by the gut bacteria from the indigestible starch and dietary fiber in the small intestine and their composition and ratios are altered in diseases associated with gut dysbiosis (Rahman et al., 2021; Shafaei et al., 2021; Kim et al., 2022).

In this study we suggested an optimized FMT donor screening and selection workflow, which included four main steps: screening of the healthy volunteers against the inclusion and exclusion criteria of the clinical study (*n* = 178) (the eligibility rate 47.8%); microbiological and microscopic stool examination (the eligibility rate 23%); blood (total blood cell count, biochemical blood analysis) and urine (urinalysis) tests (the eligibility rate 7.3%); stool SCFAs levels analysis (donor eligibility rate 2.8%) and the final FMT donor eligibility rate 1.7%.

The highest dropout rate was at the first screening step, when more than 50% of the screened for eligibility healthy volunteers could not be enrolled in the study failing to meet all the inclusion and exclusion criteria. The main reasons of failing screening process included antibiotic treatment within 6 months prior to the study, abnormal BMI and rejection to participate in the study.

It has been previously reported in the other studies that nearly half of the volunteers do not meet the inclusion criteria due to their age, medical history including antibiotic therapy shortly prior the examination and other factors. In addition, some potential stool donors experience logistical challenges or are unable to continue the screening process because of other reasons. Furthermore, some participants feel uncomfortable participating in the study due to the social stigma associated with the topic (Bénard et al., 2022; McSweeney et al., 2020).

The high dropout rate was also due to the following steps of the developed donor selection workflow, which included microbiological stool analysis, coprogram, occult blood test, blood and urine tests. The number of the eligible for FMT donors dropped from 85 to 13 donors, which corresponded to only 7.3% of initially screened donors. Most of the healthy donors were excluded at these selection steps due to the abnormal values of *Enterococcus spp*. (47.1% of healthy donors had abnormal values), total number of *Enterobacteriaceae* and *Bifidobacterium spp*. (for both parameters 41.2% of healthy donors had abnormal values) abundance. The majority of potential FMT donors who did not meet the selection criteria due to the results of the coprogram had deviations in the abundance of fatty acids (29,4%) and iodophilic flora (11,8%), indicating possible gastrointestinal tract disorders, e.g. related to insufficient exocrine pancreatic function and the diet with the excessive carbohydrate intake. Detection of occult blood in stool (*n* = 2) may serve as a marker for inflammatory bowel diseases and occult bleeding due to the erosive-ulcerative lesions of the intestinal mucosa or colorectal cancer.

Despite the high dropout rate due to the results of the stool tests, it is important to keep them in the donor selection process as the bacterial diversity of donor stool microbiota and its qualitative and quantitative characteristics are associated with the success of FMT procedure. For example, He et al., 2021 showed that the optimal microbiome composition for FMT was characterized by the predominance of *Bacteroidetes* over *Firmicutes*, low levels of *Fusobacterium* and *Ruminococcus gnavus*, high abundance of *Akkermansia muciniphila*, unclassified *Ruminococcaceae, Ruminococcus ssp*., and *Bifidobacteria* (He et al., 2021).

The metabolomic approach has been reported as valuable to assess the gut microbiota state. It was already suggested to be used as a part of the FMT donor selection process in the previous studies (Rahman et al., 2021; Shafaei et al., 2021; Olsson et al., 2021). Some of the bacterial metabolites have been already shown to impact human health, e.g. dietary tryptophane derivatives (indole and its derivatives) and SCFAs (Gold and Zhu, 2022). Indole derivatives sustain the intestinal barrier's integrity and contribute to the establishment of immune tolerance against commensal microbes (Roager and Licht, 2018; Liu et al., 2022). SCFAs, which level depends on both dietary fiber intake and diet in general, have been broadly investigated in metabolomic studies (Parada Venegas et al., 2019; Du et al., 2022; Mirzaei et al., 2021). Acetate, along with butyrate, is an important component of energy metabolism. The butyrate is produced by *Firmicutes* species mainly in the human intestine and is important for the enterocytes and has anti-inflammatory effect (Yang et al., 2023).

Therefore, we determined the SCFAs profiles within our FMT donor selection workflow to choose as eligible FMT donors the healthy volunteers with the high total stool SCFAs and butyric acid level. As a result, we selected a group of 5 FMT donors with the highest total stool SCFAs levels and the highest butyric acid level. This corresponded to 2.8% donor eligibility rate. It is important to acknowledge that the SCFA-based selection step in our workflow was exploratory in nature. The choice of the top 5 donors by total SCFA and butyrate levels was based on literature-derived associations rather than a pre-validated clinical threshold. Currently, no universally accepted reference values for ‘optimal' donor SCFA levels exist. Therefore, our approach should be viewed as a proof-of-concept for integrating metabolomics into donor screening, rather than a definitive, ready-to-implement criterion. Future prospective studies correlating donor SCFA profiles with recipient clinical outcomes are essential to establish evidence-based cutoffs and to validate the clinical utility of this approach. However, as 2 of the selected at this step donors withdrew their consent to participate in the study, the donor eligibility rate dropped to only 1.7% (final number of donors = 3).

We studied the stool microbiota composition of these 3 selected for FMT donors with 16S rRNA sequencing approach. Interestingly, among the bacteria with the highest prevalence were *Blautia* sp. SC05B48*, Gemmiger formicilis, Anaerostipes hadrus* and *Faecalibacterium_prausnitzii*. All of these bacteria are major contributors to butyrate biosynthesis (Louis et al., 2010; Sapp et al., 2022; Zhang et al., 2025) which is linked to a successful FMT outcome (Fuentes et al., 2017). This might explain the high butyric acid levels that we detected in the stool of these donors. As expected by design, the selected donors (chosen for high butyrate levels) were found to harbor a higher abundance of butyrate-producing genera, which is consistent with the intended outcome of our screening approach.

Our FMT donor selection protocol resulted in a very low donor eligibility rate (1.7%) which is lower than reported in the existing literature on similar studies. This can be explained by the stricter reference values for the microbiological analysis of stool samples (e.g. 41.2% of healthy donors were excluded due to abnormal abundance of *Bifidobacterium spp*.) or by the inclusion of SCFAs level analysis into our donor selection workflow, which has not been done in the previous studies. For example, in 2021, the largest fecal microbiota bank (OpenBiome, Cambridge, Massachusetts) reported that only 3% of potential candidates successfully passed the screening process. Paramsothy's work in 2015 on donor selection for FMT also noted that only 6-10% of screened donors were ultimately deemed suitable as FMT donors (Paramsothy et al., 2015). Serena Porcari's work in 2023 considered acceptable rates to be between 2% and 20% of potential candidates (Porcari et al., 2023).

The low FMT donor eligibility rate underscores the importance of biobanking approach. Attempts to create FMT material biobanks have been made worldwide. Biobanking projects have been launched in Switzerland, the United States, Germany to create a bank of frozen fecal samples (https://www.microbiotavault.org/). Currently, the fecal microbiota bank provides continuous access to donor fecal microbiota samples to over 750 clinics across the United States. Further development of biobanks in different countries and an easy access to the FMT donor material will aid the availability and safety of FMT treatments.

The limitation of this study is the small sample size, especially regarding the number of FMT donors who met all the selection criteria. Furthermore, the study was conducted in a single geographic/ethnic population, which may influence the microbiome composition and donor screening results. Therefore, further studies are needed to validate the developed donor selection workflow.

Nowadays there is no consensus among experts on the specific parameters of the human microbiota that contribute most to the selection process of FMT donors. Collaboration between clinical specialists, clinical laboratories, stool bank organizations, pharmaceutical companies, and regulatory agencies is essential to simplify the donor screening process and increase the effectiveness of the FMT therapy. Moreover, the standardization is currently lacking for choosing the potential healthy donor (relative of the recipient or not), donor material preparation (fresh or frozen, aerobic or anaerobic), dosage, method of administration (enemas, capsules, nasogastric tube, endoscopic delivery) and frequency of use (single or with a specific frequency). Our results suggest including quantitative SCFA analysis, particularly butyrate level, in the standard donor screening protocol for FMT. Reference values for “optimal” stool butyrate levels are to be determined in the further larger studies. Furthermore, more gut microbiota biobanks are needed to be established, which would be responsible for the search, examination, selection of donors, as well as the storage and delivery of fecal microbiota to medical organizations.

## 5 Limitations

Several limitations of this study should be acknowledged. First, the primary limitation is the lack of prospective clinical validation directly correlating our donor selection algorithm with recipient clinical outcomes. Our study establishes a screening protocol and provides a rational basis for donor selection, but it does not replace a clinical trial evaluating the efficacy of FMT using donors selected by this protocol versus standard screening.

Second, the low donor eligibility rate (1.7%) highlights the high-stringency nature of our screening but also raises concerns about scalability. While this underscores the importance of centralized stool banking, it may make national-level implementation economically challenging in some settings.

Third, our microbiome analysis was limited to 16S rRNA gene sequencing, which targets only bacteria. Archaea, fungi, and the virome were not assessed, despite their potential contributions to gut ecosystem function and FMT outcomes.

Fourth, the cross-sectional design (single stool sample per donor) does not account for temporal variability in microbiome composition, which could affect the reproducibility of donor material quality over time.

Fifth, as discussed above, the SCFA-based selection criterion was *post-hoc* and exploratory, without pre-defined cutoffs. Future studies with larger cohorts are needed to establish validated reference ranges.

Finally, our AMR screening used targeted PCR, which cannot detect novel or emerging resistance variants. Shotgun metagenomics would provide a more comprehensive assessment of the resistome and is a priority for future iterations of our protocol.

## 6 Conclusions

This study establishes a data-driven, high-stringency donor selection framework that provides a rational basis for improving FMT safety and donor quality. Our findings advocate for the standardized implementation of advanced screening technologies, particularly SCFA metabolomics, as a critical quality control step in stool banking. While clinical validation of the efficacy-enhancing potential of this protocol requires prospective studies, the strong association between butyrate-producing microbiota and favorable FMT outcomes, combined with our previous clinical observations (Seregin et al., 2024; Bespyatykh et al., 2023), supports the utility of our approach. The low donor eligibility rate (1.7%) underscores the critical importance of establishing centralized stool banks to ensure consistent access to high-quality, rigorously screened donor material.

## Data Availability

The data analyzed in this study is available in the NCBI BioProject repository (https://www.ncbi.nlm.nih.gov/bioproject) under accession number PRJNA1479977.
